# Acute effects of cardiac contractility modulation stimulation in conventional 2D and 3D human induced pluripotent stem cell-derived cardiomyocyte models

**DOI:** 10.3389/fphys.2022.1023563

**Published:** 2022-11-10

**Authors:** Tromondae K. Feaster, Nicole Feric, Isabella Pallotta, Akshay Narkar, Maura Casciola, Michael P. Graziano, Roozbeh Aschar-Sobbi, Ksenia Blinova

**Affiliations:** ^1^ Office of Science and Engineering Laboratories, Center for Devices and Radiological Health, U.S. Food and Drug Administration, Silver Spring, MD, United States; ^2^ Valo Health Inc, Alexandria Center for Life Sciences, New York, NY, United States

**Keywords:** hiPSC-CM, ECTs, stem cells, 3D microphysiological system, cardiomyocytes, engineered cardiac tissue, cardiac contractility modulation (CCM)

## Abstract

Cardiac contractility modulation (CCM) is a medical device therapy whereby non-excitatory electrical stimulations are delivered to the myocardium during the absolute refractory period to enhance cardiac function. We previously evaluated the effects of the standard CCM pulse parameters in isolated rabbit ventricular cardiomyocytes and 2D human induced pluripotent stem cell-derived cardiomyocyte (hiPSC-CM) monolayers, on flexible substrate. In the present study, we sought to extend these results to human 3D microphysiological systems to develop a robust model to evaluate various clinical CCM pulse parameters *in vitro*. HiPSC-CMs were studied in conventional 2D monolayer format, on stiff substrate (i.e., glass), and as 3D human engineered cardiac tissues (ECTs). Cardiac contractile properties were evaluated by video (i.e., pixel) and force-based analysis. CCM pulses were assessed at varying electrical ‘doses’ using a commercial pulse generator. A robust CCM contractile response was observed for 3D ECTs. Under comparable conditions, conventional 2D monolayer hiPSC-CMs, on stiff substrate, displayed no contractile response. 3D ECTs displayed enhanced contractile properties including increased contraction amplitude (i.e., force), and accelerated contraction and relaxation slopes under standard acute CCM stimulation. Moreover, 3D ECTs displayed enhanced contractility in a CCM pulse parameter-dependent manner by adjustment of CCM pulse delay, duration, amplitude, and number relative to baseline. The observed acute effects subsided when the CCM stimulation was stopped and gradually returned to baseline. These data represent the first study of CCM in 3D hiPSC-CM models and provide a nonclinical tool to assess various CCM device signals in 3D human cardiac tissues prior to *in vivo* animal studies. Moreover, this work provides a foundation to evaluate the effects of additional cardiac medical devices in 3D ECTs.

## 1 Introduction

Conventional 2D monolayer human induced pluripotent stem cell-derived cardiomyocytes (hiPSC-CMs), on stiff substrate, have been demonstrated to be useful for the evaluation of drugs and other chemical compounds (Blinova et al., 2018; Burnett et al., 2021; Yang et al., 2022). However, such models may not be appropriate for medical device optimization or safety assessment where induced effects are intrinsically reliant on more complex culture conditions, which enable a 3D level tissue response (e.g., transmural lesion formation or physiological effects). Human 3D cardiac microphysiological systems, including hiPSC-CM engineered cardiac tissues (ECTs), are gaining significant interest for cardiac safety pharmacology assessment as more biotechnology companies explore and adapt this technology (Meyer et al., 2019; Majid et al., 2020; Qu et al., 2020). The 3D ECT model has been established for the detection of known and novel inotropic compounds as well as disease modeling (Feric et al., 2019; Veldhuizen et al., 2019; Qu et al., 2020). Yet, the current gold-standard, conventional 2D monolayer hiPSC-CMs plated on stiff substrate (i.e., glass/plastic) for 7–14 days, remains popular on account of its reduced cost, high-throughput capabilities, technical ease, and numerous standardized plate-based applications. The move to more complex hiPSC-CM based models begs the question whether standard conventional 2D monolayer hiPSC-CM models, on stiff substrate, are sufficient to evaluate various cardiac contractility modulation (CCM) electrophysiological signals. Here, we do not intend to improve or develop new hiPSC-CM based models. Rather, we set out to characterize the potential utility of hiPSC-CM models to evaluate CCM device signals by comparing the performance of two well-established commercially-available models. As such, the work described here will focus on the comparison of the contractile response of conventional 2D monolayer hiPSC-CMs, on stiff substrate, and 3D ECTs to CCM.

CCM is a medical device therapy wherein non-excitatory electrical stimulations are delivered to the myocardium during the absolute refractory period (Campbell et al., 2020; Feaster et al., 2021). The first CCM device, an implantable medical device with contact leads placed in the myocardium, was approved in the U.S. in 2019 to treat heart failure (HF) patients (NYHA III), with a left ventricular ejection fraction ranging from 25 to 45% (Campbell et al., 2020; FDA 2019). Cardiac electrophysiological medical devices, including CCM and cardiac resynchronization therapy (CRT), have been developed to treat HF patients resistant to traditional pharmacotherapies. While CRT is the first-line treatment for HF patients displaying an abnormal sinus rhythm and a prolonged QRS duration, a significant population of HF patients (e.g., 60%–70%) present with normal sinus rhythm or QRS duration. CCM is indicated for such patients with prolonged QRS who are not eligible for CRT. Consequently, there is a significant gap for viable treatment options for this population and CCM is heralded as a potential solution (Campbell et al., 2020; Feaster et al., 2021). As a result, novel CCM devices are expected to be developed to address additional device functionalities and patient populations.

Lack of human nonclinical models to evaluate cardiac medical device safety and effectiveness currently hinders the regulatory review process and produces a significant burden on animal models (Harris et al., 2013; Strauss and Blinova, 2017; Feaster et al., 2021). Additionally, the direct effects of various CCM stimulation parameters on human cardiomyocyte physiology remains poorly understood. Previous studies have provided important insight into our understanding of CCM but are hindered due to species differences in cardiomyocyte biology. HiPSC-CMs are gaining interest for disease modeling, drug development, and safety pharmacology. However, their applicability for cardiac medical device assessment has not been thoroughly vetted. HiPSC-CMs are a useful *in vitro* model to assess the molecular and functional effects of CCM on human cardiac tissue (Feaster et al., 2021). However, contractile studies in hiPSC-CMs have been limited as a result of immature contractile properties when plated as conventional 2D monolayers on stiff substrates (i.e., glass or plastic) (Feaster et al., 2021; Korner et al., 2021; Huethorst et al., 2022; Narkar et al., 2022). We previously demonstrated that 2D monolayer hiPSC-CMs, on flexible substrate, displayed increased contraction and calcium handling properties when acutely stimulated with standard CCM pulse. While significant, these effects were transient and attenuated relative to that of traditional papillary muscle models (e.g., rabbit) (Brunckhorst et al., 2006). Of the variety of culture conditions tested, we discovered that the 2D monolayer hiPSC-CM model required the combination of both a submaximal extracellular Ca concentration [0.5 mM] and a flexible substrate to elucidate the CCM response (Feaster et al., 2021). Using these conditions, we demonstrated contractile amplitude and kinetic enhancement and calcium dependance (Feaster et al., 2021). However, it is important to highlight that at a physiological extracellular Ca concentration (e.g., ∼2 mM) there was no CCM contractile response (Feaster et al., 2021). To date, most hiPSC-CM studies rely on the standard conventional 2D monolayer on stiff substrate model. In such models, contractile properties are limited as a result of variable morphology, the lack of a dominant axis of myofibril alignment (Feaster et al., 2015), and a perceived glass stiffness in the GPa range (Ribeiro et al., 2015). This is contrary to 2D hiPSC-CM monolayers, on flexible substrate (i.e., Matrigel Mattress), which have an elastic modulus of approximately 5.8 kPa representing a physiologically relevant range for myocardium (i.e., 4.0–46.2 kPa) (Sun et al., 2012; Ribeiro et al., 2015).

Here, we extend these studies to acute CCM evaluation in 3D ECTs at physiological extracellular Ca concentrations. There is a significant need to understand the chronic effects of CCM (e.g., hours to days) on human biology but as a first step the acute effects must be characterized in a standardized human-based model (Narkar et al., 2022). We demonstrate that when cultured as 3D ECTs, hiPSC-CMs respond to acute electrical stimulation mimicking the standard CCM signal by an increase in contractile force. Moreover, unlike 2D monolayer hiPSC-CMs, on stiff substrate, 3D ECTs display a robust contractile response to various CCM stimulation signals. We further evaluated the complete range of clinical CCM pulse parameters in 3D ECTs and established a parameter-dependent contractile response while conventional 2D monolayer hiPSC-CMs, on stiff substrate, remained unaffected for each parameter tested. To the best of our knowledge, this is the first 3D hiPSC-CM study to elucidate the acute effects of CCM and may provide important insights on the effects of varying CCM pulse parameters at the bench, ahead of *in vivo* studies. This may improve decision making and support safety or effectiveness studies for future CCM devices. Here, we establish a standardized 3D ECT-based method to quantify and optimize acute CCM effects *in vitro*.

## 2 Materials and methods

### 2.1 2D human iPSC-CM derivation and culture

Cryopreserved hiPSC-CMs (iCell Cardiomyocytes^2^ 01434, R1017 Fujifilm Cellular Dynamics, Inc.) were thawed and plated, as previously described, according to the manufacturer’s instruction (Ma et al., 2011; Blinova et al., 2017; Blinova et al., 2018; Blinova et al., 2019). All hiPSC-CMs used in this study were derived from the same hiPSC line, which was reprogrammed from fibroblast donor tissue, isolated from an apparently healthy normal Caucasian female, <18 years old (Ma et al., 2011; Feaster et al., 2021). Briefly, 116,000 viable cells were plated per well of a 48-well glass bottom plate (MatTek) P48G-1.5–6-F on Matrigel (1:60), 356,230 Corning (Feaster et al., 2015; Hwang et al., 2015). iCell Cardiomyocytes Maintenance Medium (#M1003, Fujifilm Cellular Dynamic, Inc.) was changed every 48 h thereafter and cells were allowed to recover from cryopreservation for 7 days at 37°C before experiments were performed.

### 2.2 3D human iPSC-CM Biowire™ II tissue generation

Engineered cardiac tissues (ECTs) were generated as previously described (Feric et al., 2019; Qu et al., 2020). Briefly 100,000 hiPSC-CMs (iCell Cardiomyocytes^2^ 01434, R1017 Fujifilm Cellular Dynamics, Inc.) and 10,000 normal human ventricular cardiac fibroblasts (Lonza) were embedded in a hydrogel of fibrin (Sigma-Aldrich), collagen (Sigma-Aldrich) and Matrigel (Corning). Each well of the Biowire II platform was seeded with cell/hydrogel suspension and exposed to a 7-week electrical conditioning stimulation protocol before experiments were performed. A total of three 3D ECTs were used for each contraction experiment, with repeated measurements taken after baseline was established. The average cross-sectional area for the Biowire II platform is 0.066 ± 0.001 mm^2^ this area was used to calculate stress (Feric et al., 2019). During experiments, 3D ECTs were superfused with Tyrode’s solution as described below.

### 2.3 Electrical field (CCM) stimulation

**FIGURE 1:**
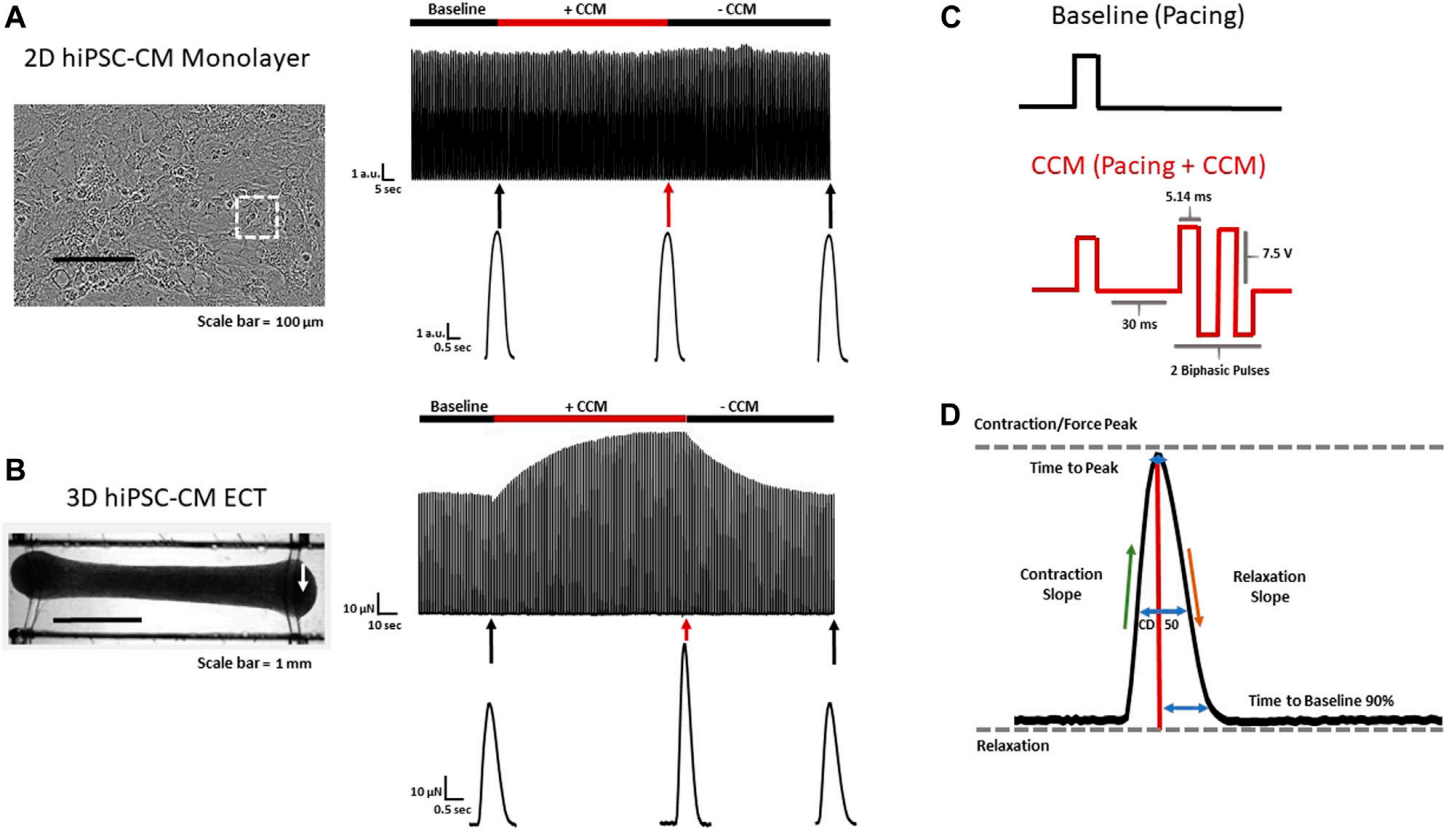
2D and 3D hiPSC-CM CCM models **(A)** Effect of standard clinical CCM signal on conventional 2D (stiff substrate) and **(B)** 3D ECT hiPSC-CM models. Representative contraction recordings before CCM (Baseline), during CCM (7.5 V) and after CCM (recovery) in 2D hiPSC-CM monolayer and 3D ECT. 3D ECTs displaying enhanced CCM-induced force. White arrow indicates edge of 3D ECT where force transducer was connected **(C)** Baseline (Control) pacing waveform (Top) and standard biphasic CCM waveform (i.e., two biphasic pulse, 7.5 V, 5.14 ms duration, 30 ms delay) (Bottom). **(D)** Schematic contractility waveform depicting key contractile parameters evaluated. Contraction slope and relaxation slope were calculated as maximum and minimum of the time derivative of the contractility amplitude respectively. White square indicates region of interest of 2D monolayer.

2D and 3D hiPSC-CM models were stimulated with commercial pulse generators: single channel (Model 4100, A-M Systems, Sequim, WA) and multi-channel (Model 3800, A-M Systems, Sequim, WA). For 2D hiPSC-CMs, custom platinum electrodes (inter electrode distance 2.0 mm, and width 1.0 mm) compatible with standard 48-well glass bottom plates (MatTek), were placed in each well sequentially, as previously described (Feaster et al., 2021). For 3D ECTs, a 600 µl chamber fabricated with parallel platinum electrodes (inter electrode distance 1.2 mm, and width 2.0 mm) was used for each tissue. In this configuration, the tissues are in-line between the parallel electrodes. For both models, pacing (i.e., baseline) and CCM electrical pulses were delivered through these platinum electrodes, resulting in field stimulation as previously described (Blinova et al., 2014) (Figure 1C). At baseline, cells were paced at 1.5 times the capture threshold using monophasic square wave pulses. 2D hiPSC-CMs were paced at 1 Hz (2 ms pacing pulse duration) and approximately 14 V/cm 3D ECTs were paced at 1 Hz (2 ms pacing pulse duration) and approximately 10 V/cm. In both models, CCM stimulation was delivered as 1 to 3 biphasic pulses, 4.5–7 ms phase duration, 1–10 V pulse amplitude, and an interphase interval of zero. The range for the delay tested, 3–160 ms, was defined as the interval between the end of the baseline pacing pulse and the beginning of the CCM pulse. All parameters evaluated (i.e., pulse delay, pulse duration, pulse amplitude, and pulse number) were determined based on the clinical device parameter range (ImpulseDynamics, 2018; ImpulseDynamics, 2019; Campbell et al., 2020) (Supplementary Table S1).

### 2.4 Measurement of contractile properties

A contractility platform and software (CellOPTIQ, Clyde Biosciences), based on video pixel displacement, was used to measure 2D hiPSC-CM contractility (Sala et al., 2018; Saleem et al., 2020b; Huethorst et al., 2022). 2D hiPSC-CMs were imaged directly in plates by an inverted fluorescence microscope (Zeiss) using a ×40 objective. For 2D hiPSC-CMs, a region of interest (ROI) was selected near the center of the well and kept constant throughout the experiment. A camera connected to the front port of the microscope was used for contraction acquisition. Temperature, 37°C, and 5% CO_2_ were maintained by an environmental control chamber (OKOLAB, Inc.). For 3D ECTs, contractile force measurements were obtained using a force transducer. Tissues were placed in a tissue bath (1 cm × 5 cm, 600 μl) and the polymer wires on one end of the tissue were cut and attached to a force transducer (AE801, Kronex Technologies, Oakland, CA) using a stainless steel wire fashioned into a basket. The other end of the tissue was immobilized using stainless steel wires attached to a micromanipulator (Supplementary Figure S1A; Supplementary Video S1). The AE801 is a silicon-based strain gauge with two piezoresistive elements. The AE801 was connected to a Wheatstone bridge amplifier in half bridge mode (Transbridge 4M, WPI, Sarasota, FL) which converts the resistance changes in the strain gauge to a voltage signal. The AE801 was pre-calibrated prior to experimentation with known weights and a relationship between voltage and force of 104.4 μN/mV was used to convert the voltage recording to force measurements. Signals were digitized using the Digidata 1322 A and recorded at 10 kHz with Axoscope Software (Molecular Devices, San Jose, CA). Tissues were constantly superfused in the tissue bath with Tyrode’s solution at 4 ml/min and the temperature in the bath was maintained at 37°C.

All experiments were performed in Tyrode’s solution containing (in mmol/L): CaCl_2_ 1.8, NaCl 134, KCl 5.4, MgCl_2_ 1, glucose 10, and HEPES 10, pH adjusted to 7.4 with NaOH at 37 °C. To evaluate extracellular Ca concentration effects on CCM, we adjusted total extracellular Ca range from 0.5 to 4 mM (Feaster et al., 2015; Feaster et al., 2021). For each experimental group, a minimum 5 s recording was taken and analyzed, as previously described (Feaster et al., 2015; Feaster et al., 2021). The contractile baseline was established by allowing equilibration to a steady state before measurements. Contractile properties, including contraction amplitude or force, time to peak (the time from 10% peak height to the peak of contraction), time to baseline at 90% (the time from peak of contraction to baseline 90% of peak height), contraction duration at 50%, CD 50% (the time from 50% peak height to peak of contraction and from peak to 50% peak height of relaxation), contraction slope, and relaxation slope were evaluated (Figure 1D, Supplementary Table S2) Contraction slope and relaxation slope were calculated as the maximum and minimum time derivative of the contractility amplitude, respectively.

### 2.5 Statistical analysis

All statistical analyses were performed using GraphPad Prism 8 software (Prism 8, GraphPad Software, CA). Differences among the groups are presented as mean ± standard error of the mean (SEM). Differences were assessed as fold-change relative to baseline (i.e., pacing only) using a Two-Way ANOVA. Results were considered statistically significant if the *p*-value was less than 0.05, adjusted by Tukey correction for multiple comparisons.

## 3 Results

### 3.1 Comparison of 2D and 3D human CCM models

**FIGURE 2:**
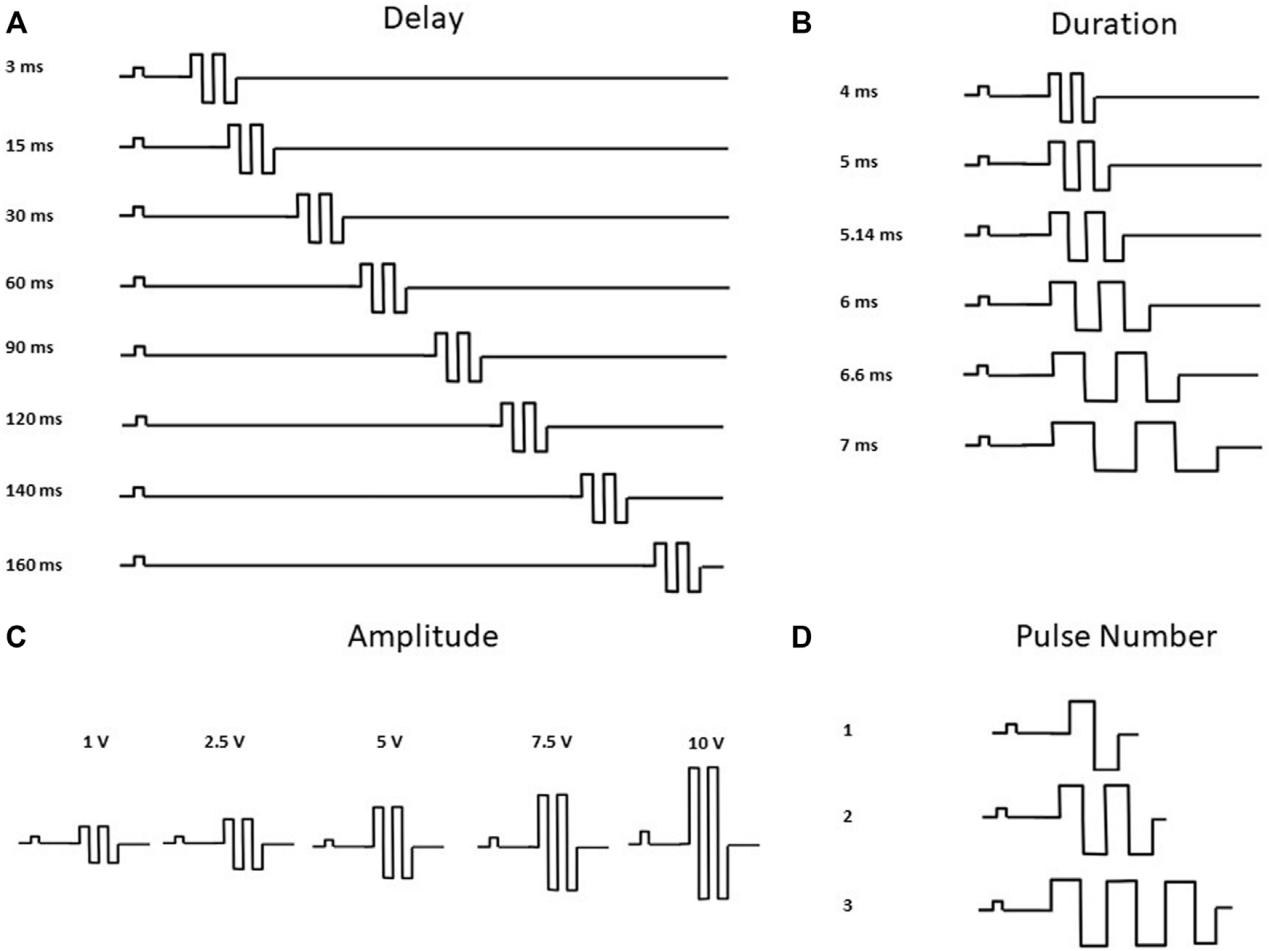
CCM Pulse Waveforms Evaluated. Waveforms depicting CCM Parameters Tested **(A)** Pulse Delay 1–160 ms, additional parameters fixed at duration 5.14 ms, amplitude 7.5 V and pulse number 2. **(B)** Pulse Duration 4.5–7 ms, additional parameters fixed at delay 30 ms, amplitude 7.5 V and pulse number 2 **(C)** Pulse Amplitude 1–10 V, additional parameters fixed at delay 30 ms, duration 5.14 ms and pulse number 2. **(D)** Pulse Number 1 to 3 Pulses, additional parameters fixed at delay 30 ms, duration 5.14 ms and amplitude 7.5 V. Pulse waveforms not to scale (ImpulseDynamics, 2018; ImpulseDynamics, 2019).

Commercially available, cryopreserved hiPSC-CMs were evaluated as conventional 2D monolayers, on stiff substrate (i.e., glass) (Feaster et al., 2015; Wang et al., 2018), and as 3D ECTs (Figure 1A,B). For consistency and physiological relevance, an extracellular calcium concentration of 1.8 mM was used for both models. We first evaluated the effects of the standard acute CCM stimulation parameters in both hiPSC-CM models: two biphasic pulses, 7.5 V, 5.14 ms duration, and 30 ms delay (Figure 1C) (Stix et al., 2004; Kuschyk et al., 2017; Tint et al., 2019; Mastoris et al., 2021; Amiraslanov et al., 2022). Significantly enhanced contractile properties were observed in 3D ECTs including increased amplitude (i.e., force) and accelerated contraction and relaxation slopes (Figure 1B) (Table. 1). Conventional 2D hiPSC-CM monolayers, displayed no CCM-induced contractile response (Figure 1A) (Table. 1). Herein, we measure the acute effects of various clinical CCM “doses” (Figure 2) (Supplementary Table S1) on cardiac contractile properties (e.g., amplitude, time to peak and contraction slope) (Figure 1D) compared to that of baseline (i.e., standard field stimulation pacing, 1 Hz) (Figure 1C), to determine which model can best evaluate the effects of various CCM signals *in vitro*.

**TABLE 1:** Standard CCM signal contractile characterization.

Parameter	2D monolayer hiPSC-CM CCM	3D ECT CCM
Peak Amplitude/Force	−1% ± 0.01%	43 ± 3%*
Contraction Duration 50%	1% ± 0.02%	9 ± 2%*
Contraction Slope	3% ± 0.03%	25 ± 2%*
Relaxation Slope	−2% ± 0.02%	50 ± 3%*
Time to Peak	1% ± 0.01%	16 ± 1%*
Time to Baseline 90%	−1% ± 0.01%	0 ± 2%
N	16^a^	12^b^

^a^ 16 independent wells

^b^ 3 total ECTs, 12 repeated measurements

### 3.2 CCM is sensitive to extracellular calcium modulation in 3D ECTs

**FIGURE 3:**
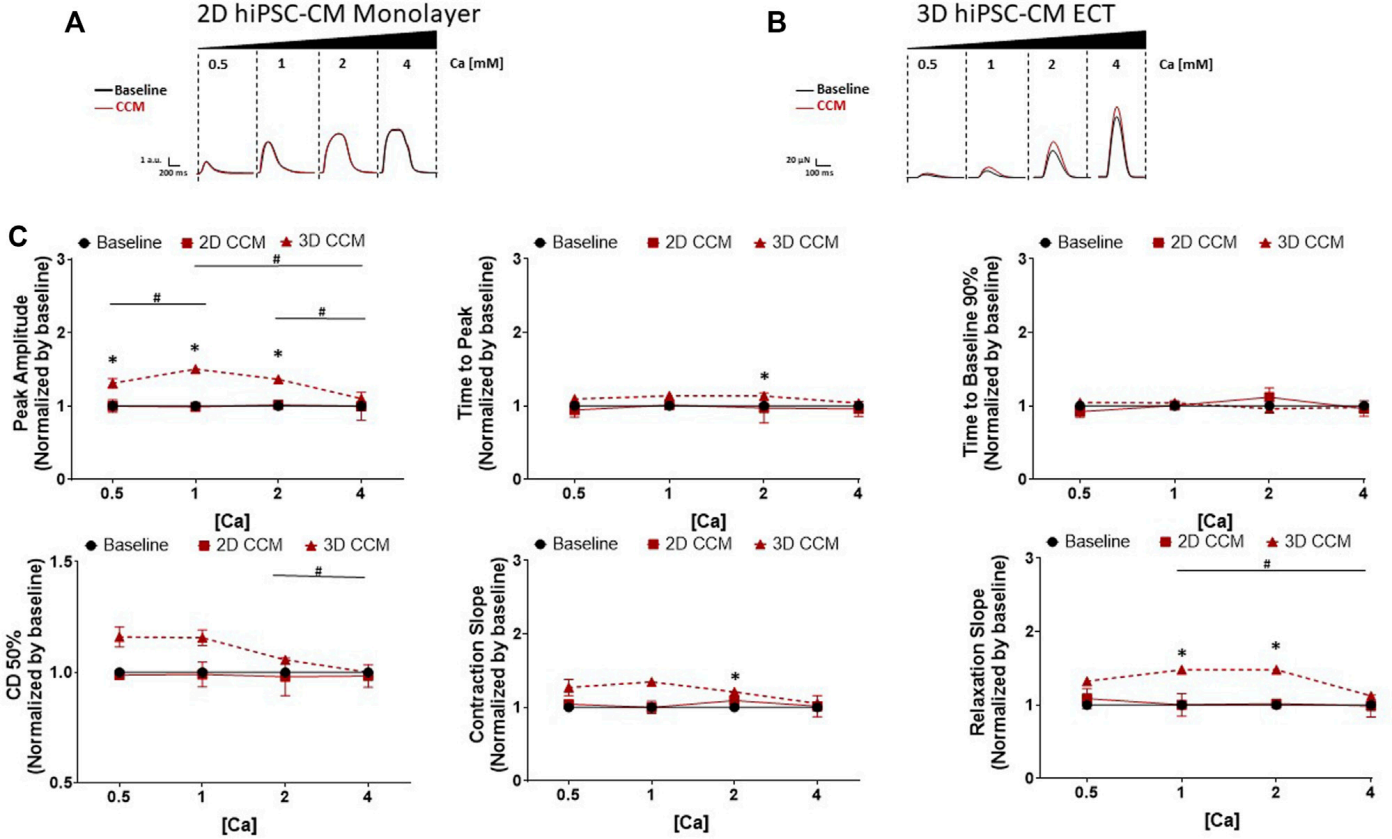
Effect of Extracellular Ca Modulation on CCM Response in 2D and 3D models **(A)** Representative contraction traces for baseline (i.e., field stimulation pacing, 1 Hz). and CCM for 2D hiPSC-CMs monolayer and **(B)** 3D ECTs. CCM was applied as the standard biphasic CCM waveform (i.e., two biphasic pulse, 7.5 V, 5.14 ms duration, 30 ms delay). hiPSC-CMs were exposed to increasing concentrations of extracellular Ca [Ca_o_] 0.5–4 mM **(C)** Summary data graphs. Data are mean ± SEM. *n* = 3 – 6 per group. ^*^ *p* < 0.05 (3D vs. BL), ^#^ *p* < 0.05 (3D vs. 3D), ^+^ *p* < 0.05 (2D vs. BL).

To determine the CCM dependance on extracellular Ca concentration, we evaluated the effects of CCM as a function of increasing levels of extracellular calcium concentration from 0.5 to 4 mM. Using the standard CCM stimulation settings (Figure 1C), 3D ECTs displayed significantly enhanced contractile amplitude (Figure 3B,C) relative to baseline at 0.5, 1 and 2 mM Ca. 2D monolayer hiPSC-CMs, on stiff substrate, displayed no CCM-induced response at any Ca concentration tested relative to baseline (Figures 2A,C). Consistent with previous studies, the 3D ECT response to CCM was blunted at higher extracellular calcium concentrations (Burkhoff et al., 2001; Brunckhorst et al., 2006; Feaster et al., 2021) (Figures 3B,C). The CCM-induced increase in 3D ECT contractile force was significantly more pronounced when extracellular calcium concentration was lowered from 4 to 2 mM. These results suggest that the CCM effects on contractile force in 3D ECTs, including increased amplitude and accelerated contraction and relaxation slopes, are dependent on the extracellular free Ca concentration.

### 3.3 CCM pulse delay enhances contractile properties

**FIGURE 4:**
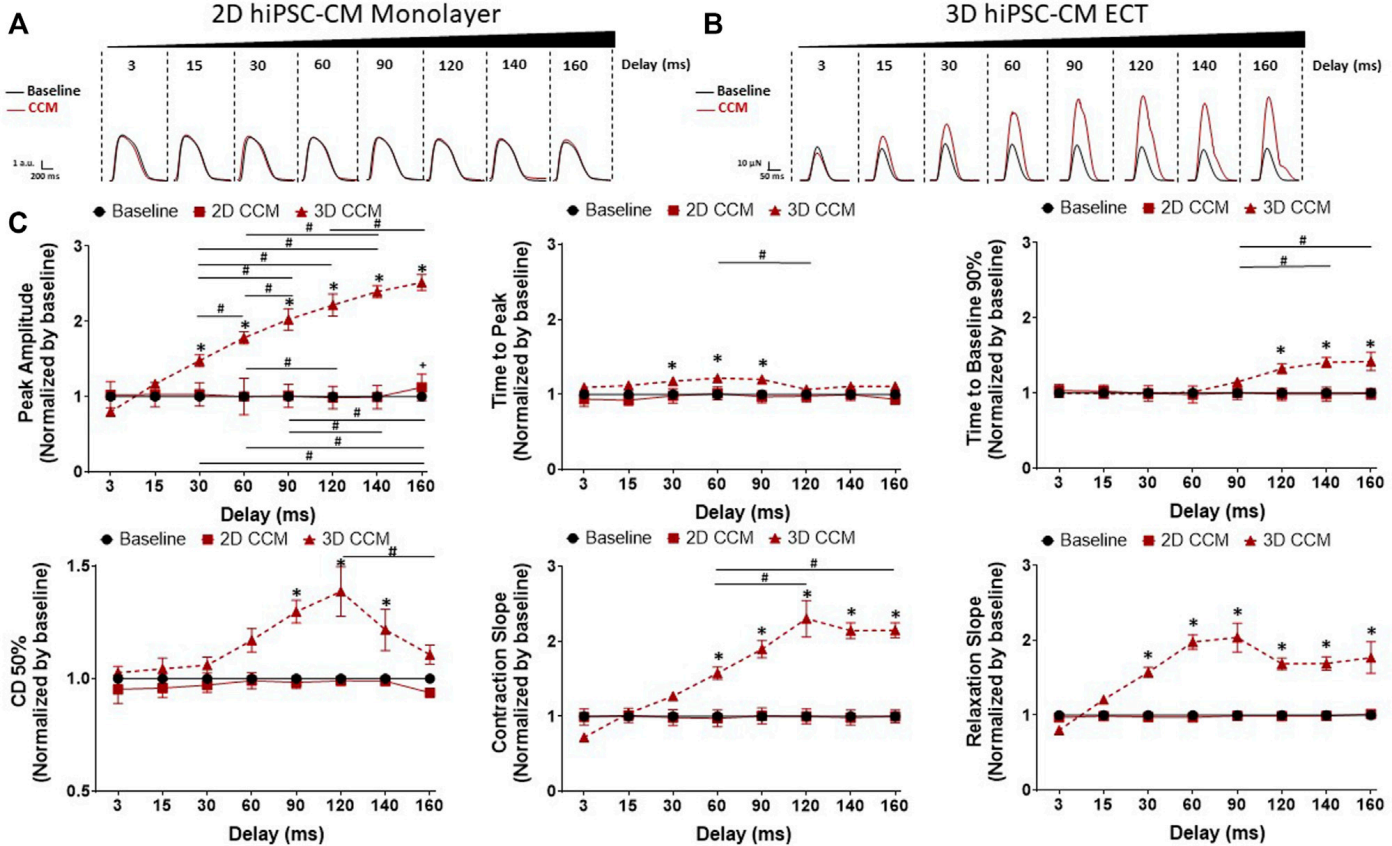
Effect of CCM pulse delay on 2D and 3D hiPSC-CM contractile properties **(A)** Representative contraction traces for baseline (i.e., field stimulation pacing, 1 Hz). and CCM for 2D monolayer hiPSC-CMs and **(B)** 3D ECTs **(C)** Summary data graphs. Data are mean ± SEM. n = 3–6 per group. ^*^ *p* < 0.05 (3D vs. BL), ^#^ *p* < 0.05 (3D vs. 3D), ^+^ *p* < 0.05 (2D vs. BL).

We next evaluated the effects of varying the duration of the delay between pacing and CCM stimulation on 2D and 3D hiPSC-CM models (Figure 2A). The range of delays tested was comparable to that of the clinical device (i.e., 3–160 ms). 3D ECTs exposed acutely to clinically relevant CCM pulse parameters exhibited enhanced cardiac contractility that subsided gradually when the CCM signal was eliminated. Specifically, 3D ECTs displayed enhanced contractile properties as a function of CCM delay timing including increased force amplitude and accelerated contraction and relaxation slopes (Figures 4B,C). Enhanced contractile force was observed with ≥30 ms delay. At the shortest delay tested (3 ms), 3D ECTs displayed a negative inotropic response that was reversed in a time dependent-manner as the pulse delay time increased into the refractory period. At the longest delay tested (160 ms), 3D ECTs displayed an increased contraction amplitude of 115.8 ± 11.4 µN (1.75 ± 0.17 mN/mm^2^) (Supplementary Table S3). Likewise, contraction and relaxation slopes were also slowed at 3 ms but accelerated in a time dependent manner as the pulse delay time increased. At the longer delays tested (120–160 ms), there was a significant prolongation of the contraction duration 50% and time to baseline 90%. For each CCM pulse delay investigated, 2D monolayer hiPSC-CMs, on stiff substrate, displayed a negligible CCM-induced response (Figures 4A,C). Taken together these data demonstrate that varying the CCM pulse delay of the acute CCM stimulation affects human 3D ECT contractile properties *in vitro* while 2D monolayer hiPSC-CMs, on stiff substrate, have a negligible contractile response.

### 3.4 CCM pulse duration increases contractile properties

**FIGURE 5:**
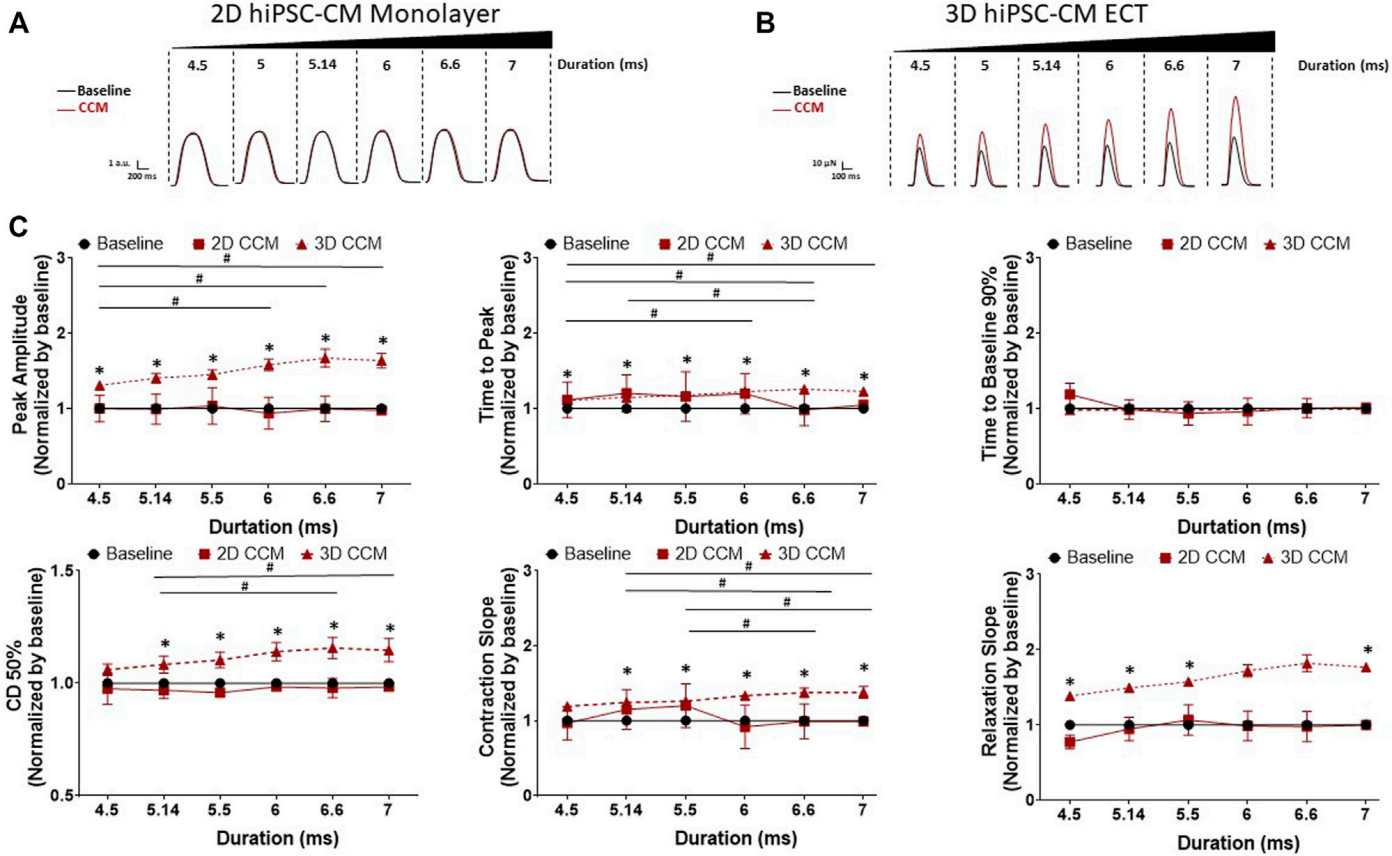
Effect of CCM pulse duration on 2D and 3D hiPSC-CM contractile properties **(A)** Representative contraction traces for baseline (i.e., field stimulation pacing, 1 Hz). and CCM for 2D monolayer hiPSC-CMs and **(B)** 3D ECTs **(C)** Summary data graphs. Data are mean ± SEM. *n* = 3–6 per group. ^*^ *p* < 0.05 (3D vs. BL), ^#^ *p* < 0.05 (3D vs. 3D), ^+^ *p* < 0.05 (2D vs. BL).

To assess the acute effects of CCM pulse duration time on human cardiomyocyte contractility, we evaluated various clinical CCM pulse durations from 4.5 to 7 ms (Figure 2B). 3D ECTs displayed a significantly increased inotropic response as a function of increasing CCM pulse duration relative to baseline (Figures 5B,C). Similarly, contraction and relaxation slopes were enhanced as CCM pulse duration increased (Figures 5B,C). At the longest duration tested (7 ms), 3D ECTs displayed increased contraction amplitude of 80.8 ± 9.2 µN (1.22 ± 0.14 mN/mm^2^) (Supplementary Table S3). Varying the CCM pulse duration resulted in the widening of the contraction duration 50% at ≥ 5.14 ms. 2D monolayer hiPSC-CMs, on stiff substrate, displayed no CCM-induced response at any of the pulse durations tested (Figures 5A,C). 3D ECT effects remained for the entire duration of CCM stimulation and returned to baseline when the CCM signal was eliminated. These results suggest that increasing the CCM pulse duration induces a “dose” dependent increase in the contractile properties of 3D ECTs.

### 3.5 CCM pulse amplitude modulates contractile properties

**FIGURE 6:**
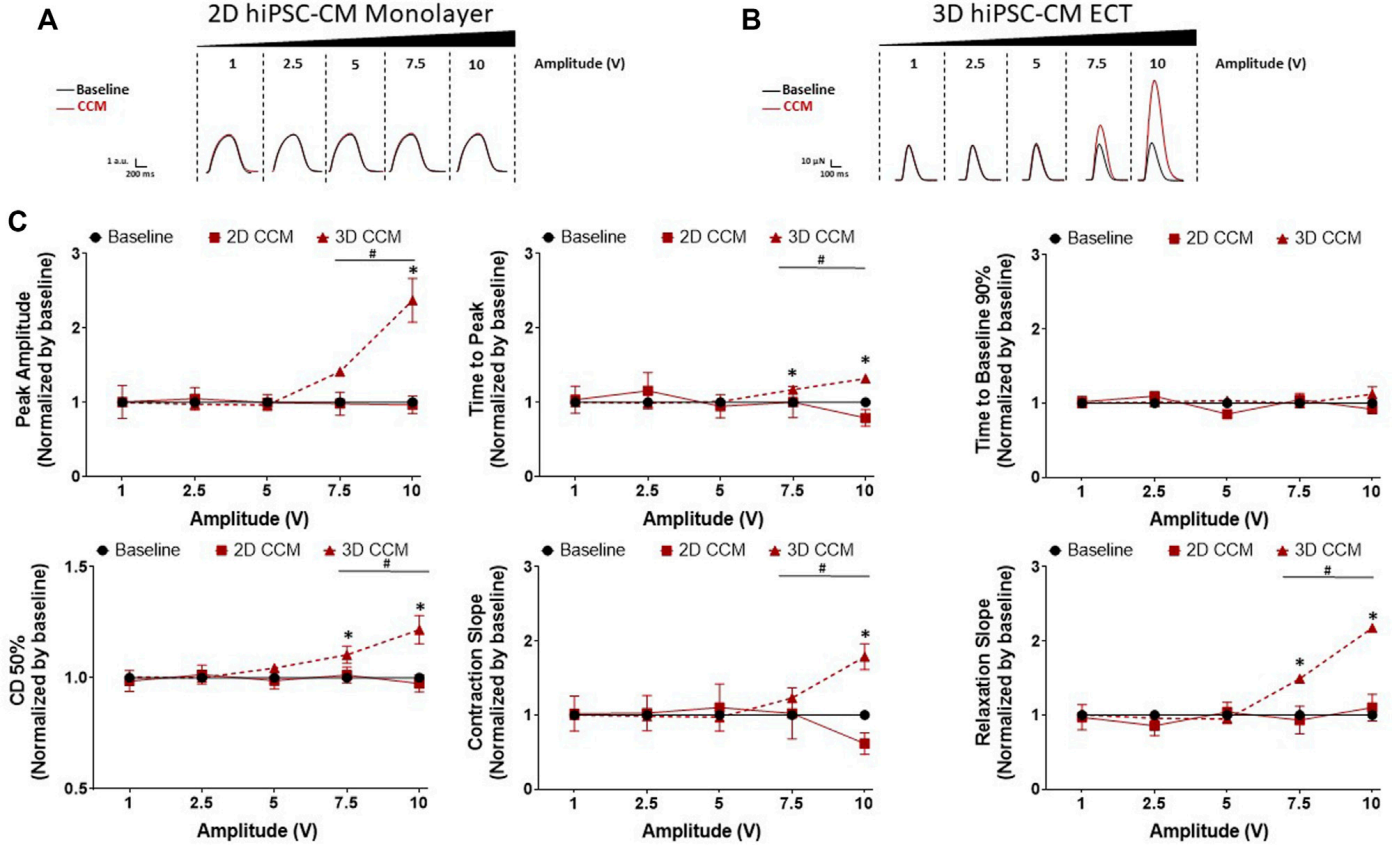
Effect of CCM pulse amplitude on 2D and 3D hiPSC-CM contractile properties **(A)** Representative contraction traces for baseline (i.e., field stimulation pacing, 1 Hz). and CCM for 2D hiPSC-CMs monolayer and **(B)** 3D ECTs **(C)** Summary data graphs. Data are mean ± SEM. *n* = 3–6 per group. ^*^ *p* < 0.05 (3D vs. BL), ^#^ *p* < 0.05 (3D vs. 3D), ^+^ *p* < 0.05 (2D vs. BL).

Next, we investigated the dependence of human cardiac contractile properties on acute CCM amplitude (i.e., voltage), from 1 to 10 V, in 2D and 3D hiPSC-CMs models (Figure 2C). HiPSC-CMs were stimulated with various CCM amplitudes. We found increasing the CCM amplitude resulted in a significantly increased contraction amplitude in 3D ECT relative to baseline (Figures 6B,C). Additionally, contraction and relaxation slopes were accelerated, and contraction duration 50% widened at higher voltages in 3D ECTs. At the highest amplitude tested (10 V), 3D ECTs displayed increased contraction amplitude of 111.8 ± 5.8 µN (1.69 ± 0.08 mN/mm^2^) (Supplementary Table S3), whereas 2D hiPSC-CMs displayed no CCM-induced response (Figures 6A,C). These results suggest that increasing the CCM amplitude produces a voltage-dependent increase in contractile force and accelerated contraction and relaxation slopes in 3D ECTs.

### 3.6 CCM pulse number augments 3D ECT contractile properties

**FIGURE 7:**
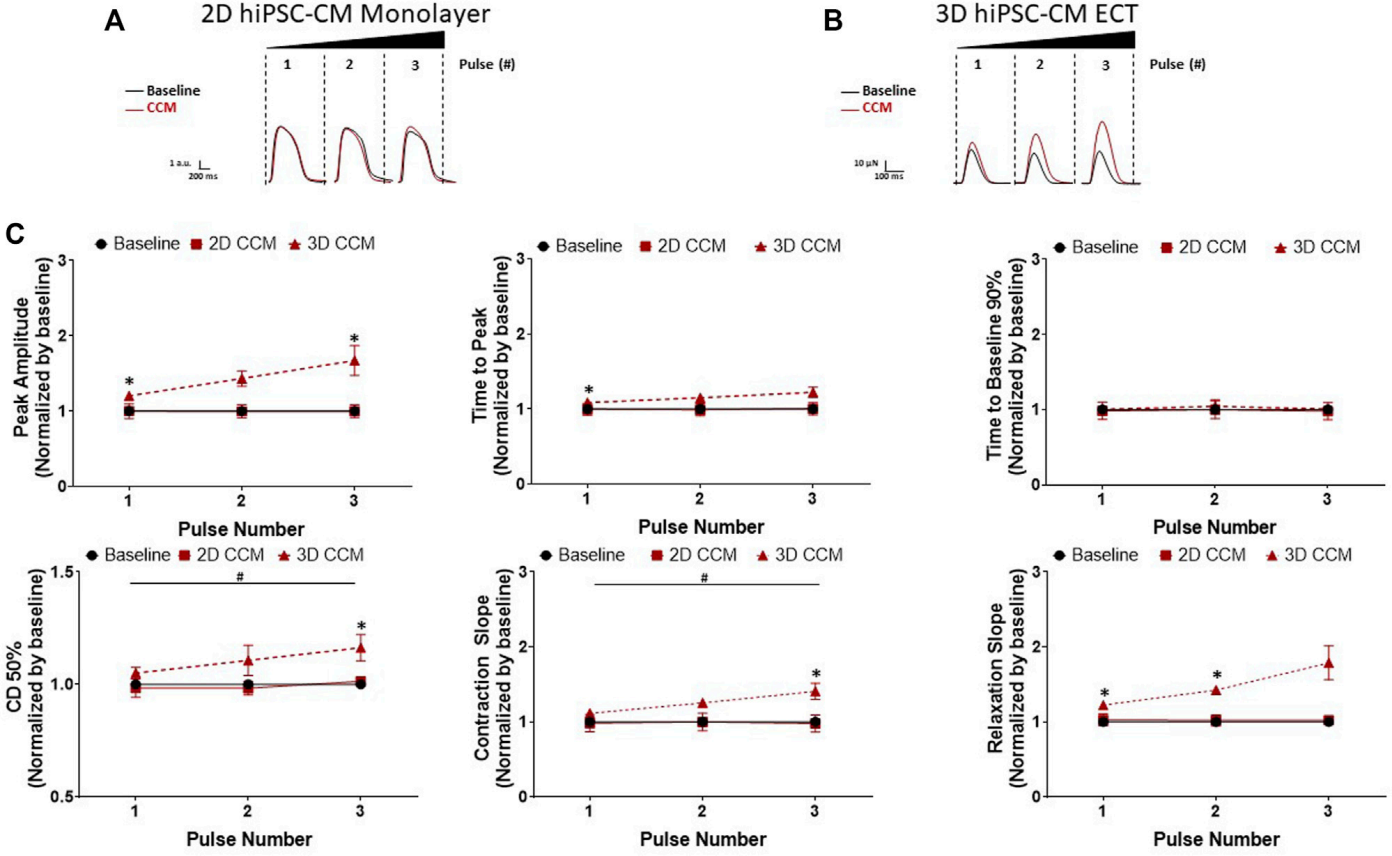
Effect of CCM pulse number on 2D and 3D hiPSC-CM contractile properties **(A)** Representative contraction traces for baseline (i.e., field stimulation pacing, 1 Hz). and CCM for 2D monolayer hiPSC-CMs and **(B)** 3D ECTs **(C)** Summary data graphs. Data are mean ± SEM. *n* = 3–6 per group. ^*^ *p* < 0.05 (3D vs. BL), ^#^ *p* < 0.05 (3D vs. 3D), ^+^ *p* < 0.05 (2D vs. BL).

To investigate the effect of pulse number on the 2D and 3D hiPSC-CMs models, pulse number was increased from 1 to 3 pulses. 3D ECTs displayed a CCM pulse number dependent (Figure 2D) increase in contraction amplitude from 1 to 3 pulses (Figures 7B,C). Additionally, we observed significantly accelerated contraction and relaxation slopes and contraction duration 50% prolongation as a function of CCM pulse number (Figures 7B,C). At the highest pulse number tested (3 pulses), 3D ECTs displayed an increased contraction amplitude of 76.6 ± 5.2 µN (1.16 ± 0.08 mN/mm^2^) (Supplementary Table S3). On the other hand, 2D hiPSC-CMs displayed a negligible response to varying the number of CCM pulses (Figures 7A,C). These results demonstrate that 3D ECTs respond to increased CCM pulse number by increased peak contractile force and accelerated contraction and relaxation slopes.

## 4 Discussion

### 4.1 2D and 3D hiPSC-CM CCM models

In this study, we establish a robust *in vitro* method to quantify and optimize the effect of various CCM stimulation parameters in 3D ECTs to improve device developers decision-making capabilities. CCM is a cardiac therapy approved for HF patients with reduced ejection fraction. However, the ways in which various CCM signal parameters affect human cardiomyocyte contractile properties have not been completely defined *in vitro*. Video-based and force-based analyses were used to quantify the effects of a range (i.e., “doses”) of clinical electrical CCM parameters on human cardiomyocyte contractility (e.g., force) in conventional 2D hiPSC-CMs, on stiff substrate, and 3D hiPSC-CM (ECTs) models. We demonstrated that 3D ECTs responded to various CCM signals with an increased peak contractile force in a pulse parameter-dependent manner. Moreover, to our knowledge, we have for the first time quantified the acute effects of CCM pulses on a 3D microphysiological system comprised of multiple cardiac cell types, specifically cardiomyocytes and cardiac fibroblasts. Here, utilizing a 3D ECT model, we demonstrate a sustained augmented CCM-induced contractility response, at physiological Ca concentrations (1.8 mM). On the other hand, standard 2D monolayer hiPSC-CMs, on stiff substrate, that were cultured for 7 days display no contractile response to CCM. The discrepancy between 2D monolayer hiPSC-CMs models and 3D ECTs is likely the result of electrical conditioning, time in culture, uniaxial tension, and the contribution of non-cardiomyocytes (i.e., cardiac fibroblast) (Eng et al., 2016; Ronaldson-Bouchard et al., 2018).

### 4.2 Various CCM electrical signals modulate 3D ECT function

Clinically CCM has been associated with reduced HF hospitalization and improved quality of life (Campbell et al., 2020). Several studies have demonstrated an increased ejection fraction and accelerated dP/dt_max_ (i.e., maximum systolic upslope) with CCM treatment (Pappone et al., 2002; Lawo et al., 2005; Nagele et al., 2008). However, how various CCM parameters affect human cardiomyocyte biology is largely unknown. To understand how different CCM signal parameters affect cardiac contractility, we investigated the effects of various CCM pulse signals on human cardiac function *in vitro*. We found that various CCM parameters (amplitude, duration, delay, and pulse number) enhanced the contractile force of 3D ECTs in a parameter-dependent manner. Specifically, we demonstrate a parameter-dependent acceleration of contraction and relaxation slopes that is consistent with an accelerated dP/dt_max_. Additionally, we observed a pulse number-dependent increase in contraction amplitude in 3D ECTs. By setting the total pulse duration time to a fixed interval, we demonstrated that these effects were likely the result of an increase in total duration time rather than absolute number of CCM pulses (Supplementary Figure S2). This is consistent with a comparable electric field (E) for 1 to 3 CCM pulses when total duration was fixed. One potential explanation of this could be prolonged channel activation by L-type calcium channels or intracellular SR calcium stores as the total CCM stimulation duration is increased. When evaluating CCM pulse delay we found the shortest delay between pacing and CCM pulse investigated (3 ms) resulted in reduced contractile amplitude, and a pulse delay of 120 ms induced a prolongation of contraction duration 50% and time to baseline 90%. Conversely, we did not investigate the effects of CCM pulse delay on action potential morphology, or if this translates to prolongation or triangularization of the action potential, as it was outside the scope of this work. However, it is important to note that significant prolongation or triangularization of the action potential may indicate a possible safety liability (Blinova et al., 2018; Gintant et al., 2020) or a proarrhythmic substrate. We have previously investigated patient-specific responses in 2D hiPSC-CMs, setting the stage for such a comparison in 3D ECTs. We compared clinical drug concentration-dependent QT prolongation with *in vitro* drug concentration-dependent action potential duration prolongation (Blinova et al., 2019). In the future, 3D ECT data may be leveraged to better understand CCM in patient-specific populations or cohorts using disease-specific models (e.g., clinical trial in a dish) (Strauss and Blinova, 2017; Fermini et al., 2018; Blinova et al., 2019), and to identify potential CCM super responders (Al-Ghamdi et al., 2017; Hesselson et al., 2022) before therapy is needed. Likewise, these data provide an *in vitro* tool to optimize CCM parameters and tailor said parameters to an individual patient using patient-specific 3D ECTs following clinical presentation.

### 4.3 Comparison of *in vitro*, *ex vivo*, and *in vivo* CCM studies

Previous nonclinical CCM studies are challenging to correlate with each other because they apply a variety of CCM parameters, models, and species (Feaster et al., 2021). Consequently, there are conflicting reports of the effects of CCM on human CM contractility (Campbell et al., 2020; Burkhoff et al., 2001; Winter et al., 2014). Likewise, clinical translation of such results is complex. Despite this, the consensus is that CCM stimulation increases contractility, calcium handling, and enhances gene expression without negatively affecting mitochondrial function. One *in vitro* study using an isolated rabbit papillary muscle model demonstrated enhanced contractility in a manner dependent on the CCM pulse parameters (e.g., amplitude polarity) (Brunckhorst et al., 2006). However, this study used nonclinical CCM pulse parameters and a nonhuman animal model. We previously demonstrated CCM induced enhanced contractility and calcium handling using *in vitro* isolated rabbit CMs and 2D hiPSC-CMs, on flexible substrate (Blinova et al., 2014; Feaster et al., 2021). Still, the maximum response was transient in these two studies. In 2D hiPSC-CMs, on flexible substrate, a submaximal extracellular Ca concentration of 0.5 mM was necessary to reveal the CCM contractile response. Here, our goal was to use a physiological extracellular calcium concentration of 1.8 mM. However, in the presence of submaximal extracellular calcium concentrations, 3D ECTs maintained a superior contractile response relative to 2D hiPSC-CMs, on flexible substrate (Supplementary Figure S3). Several *ex vivo* whole heart ferret models demonstrated that CCM induced increased force and calcium handling as well as calcium dependance (Burkhoff et al., 2001; Mohri et al., 2002; Mohri et al., 2003). Similarly, an *ex vivo* whole heart rabbit model demonstrated increased contraction and shortened monophasic action potential duration along with a dependance on β-adrenergic signaling (Winter et al., 2014; Winter et al., 2011). We previously demonstrated, in an *ex vivo* whole rat heart model, that CCM enhanced left ventricular pressure and β-adrenergic signaling contributed to the CCM response (Blinova et al., 2014). A multitude of *in vivo* canine models (i.e., failing heart) demonstrate improved left ventricular function (enhanced ejection fraction) induced by CCM. While these seminal papers provide important insight into the effects and mechanisms of CCM, these studies are costly, time consuming, and rely heavily on large and small animal models (Sabbah et al., 2001; Mohri et al., 2002; Morita et al., 2003). As such, a robust human-based model to reproducibly evaluate CCM signals *in vitro* is needed to aid the development of novel devices and understand the effects of various signals on human cardiomyocyte biology. In this work we demonstrate the first nonclinical CCM study using the approved clinical range of CCM parameters (Campbell et al., 2020) in a human microphysiological system (3D ECTs). Although other methods, such as traditional papillary muscle models (e.g., rabbit) and *in vivo* canine models are amenable to CCM assessment, our 3D ECT method enables CCM evaluation in human cardiac tissue, ahead of costly animal testing, significantly assisting the 3Rs initiative (Schechtman, 2002). This 3D ECT CCM method can serve as a foundation for the development and optimization of novel cardiac medical devices and can be multiplexed to evaluate CCM effects on additional cardiac excitation-contraction coupling (E-C) readouts including electrophysiology (i.e., action potential) and calcium handling.

### 4.4 Study limitations

We recognize there are inherent differences in 2D and 3D models including time in culture and inclusion of cardiac fibroblasts. To ensure the most accurate comparison of conventional 2D monolayer hiPSC-CMs, on stiff substrate, to 3D ECTs, experimental conditions were unified in a number of important ways including: 1) selection of a physiological extracellular calcium concentration of 1.8 mM (Yee, 2008), an accepted standard for *in vitro* experiments (Saleem et al., 2020a; Bartolucci et al., 2020; Tsan et al., 2021); 2) we used commercially available hiPSC-CMs from the same manufacture in both 2D and 3D models as well as comparable cell numbers for each 2D well and 3D ECT; 3) platinum electrodes were used in both systems to limit corrosion potential; 4) both 2D and 3D experiments were conducted at a physiological temperature. However, our study has several limitations. For example, conventional 2D monolayer hiPSC-CMs, on stiff substrate, display several features of immature cardiomyocytes including spontaneous beating. As a standard, conventional 2D hiPSC-CMs are routinely cultured for 7–14 days without electrical conditioning ahead of experiments. However, there are frequently stimulated during experimentation to eliminate potential rate-dependent effects (Patel et al., 2019). 3D ECTs were cultured for approximately 7 weeks with the addition of electrical conditioning. As such, we cannot exclude the impact of long-term culture or electrical conditioning on the 3D ECT CCM response. While technically feasible, culturing 2D monolayers for 7 weeks is not trivial and the nature of the 2D environment does not provide the optimal conditions for long-term pacing. In conventional 2D culture, unstable extracellular matrix and monolayer integrity are of prime concern. On the other hand, 3D ECTs benefit from supporting cells and a stable 3D environment, as the cells are embedded in the extracellular matrix gel. 3D ECTs represent a functionally enhanced hiPSC-CM model with intact isoproterenol-induced positive inotropy, a positive force-frequency, and post-rest potentiation, which likely augmented the CCM contractile response and enabled the shift in the experimental conditions to a physiologic extracellular Ca concentration (Feric et al., 2019). Direct force measurements were used for 3D ECTs for the evaluation of contraction amplitude. In conventional 2D monolayer hiPSC-CMs, on stiff substrate, video-based pixel displacement was used to measure contractile properties due to a lack of cellular anisotropy and the limited cellular movement (i.e., shortening) of the model. While both contraction amplitude measurements (i.e., force and displacement) typically have a synergistic relationship, it is conceivable for contractile force to increase while cellular movement reduces or remains neutral as is the case for isometric contraction forces. Both 2D and 3D models lack a neuronal component necessary to elucidate the contribution of sympathetic stimulation through cardiac ganglion. Toward this goal, we are actively investigating the contribution of hiPSC-neurons to the CCM response (Narkar et al., 2022). Moreover, the contribution of non-cardiomyocytes (i.e., cardiac fibroblast) cannot be overlooked as well as the mixed population of hiPSC-CMs from each cardiac subtype (i.e., ventricular, atrial, and nodal) represented in both 2D and 3D models used here. Additionally, the commercial hiPSC-CMs used here represent an apparently ‘healthy’ cardiac model whereas CCM is indicated for HF patients. These models will be extended to diseased backgrounds including HF, DCM, and HCM. However, as a first step in this direction, demonstration of a CCM response on healthy cells is required. Clinical translation and the correlation of these data with human patient outcomes is of significant interest but is currently hindered due to limited access to human clinical data. In this study we focused primarily on the currently approved clinical range of CCM parameters. As such, we did not investigate minimum and maximum response. The CCM parameter range evaluated here was selected to span that of the clinical device capabilities where applicable. Experiments to test combinations of the most promising parameters that yield maximal contractile response with minimal pathological consequences are ongoing in our laboratories. Clinically the beneficial effects of CCM are achieved following prolonged stimulation and have been suggested to be related to slow tissue remodeling which is outside of the scope of this acute study (Butter et al., 2008).

## 5 Conclusion

This work lays the foundation for an *in vitro* CCM parameter evaluation tool and may support safety or effectiveness studies for future CCM devices as well as other cardiac electrophysiological medical devices in general. Here, we demonstrate several important findings. 1) 3D ECTs respond to acute clinical CCM stimulation parameters at physiological Ca concentrations. 2) 3D ECTs respond to the changes in various clinical CCM stimulation parameters (i.e., pulse delay, pulse duration, pulse amplitude, and pulse number), as a function of each parameter, by an increase in contractile force. This provides a nonclinical model to test and optimize various non-excitatory electrical signal parameters and combinations. 3) 3D ECTs display accelerated contraction and relaxation slopes when stimulated with CCM, which is consistent with an accelerated dP/dt_max_ and may be beneficial in the context of systolic or diastolic dysfunction. 4) Conventional 2D monolayer hiPSC-CMs, on stiff substrate (e.g., glass/plastic), and cultured for 7 days do not respond to CCM. 5) CCM pulse number had a negligible effect on contractile response in human cardiomyocytes unlike the total pulse duration time, which was the driving factor for enhanced contractile force in situations where pulse number was increased. 6) In 3D ECTs, the CCM response is sensitive to changes in the extracellular Ca concentration resulting in a blunted effect at higher concentrations (i.e., 4 mM). Taken together, the current study demonstrated that the 3D ECT model can recapitulate CCM-induced contractility increase, consistent with the model being predictive of the effects of electrophysiological stimulation on human tissue. Thus, there is a significant need to evaluate the effects of additional cardiac electrophysiological medical devices (e.g., ablation, CRT, or ICD) in human models such as 3D ECTs. Toward that goal, we are actively evaluating cardiac medical devices in a variety of novel 3D *in vitro* hiPSC models to address regulatory science knowledge gaps.

## Data Availability

The raw data supporting the conclusion of this article will be made available by the authors, without undue reservation.

## References

[B1] Al-Ghamdi B. Shafquat A. Mallawi Y. (2017). Cardiac contractility modulation therapy: Are there superresponders? Hear. Case Rep. 3, 229–232. 10.1016/j.hrcr.2017.02.004 PMC5419822 28491808

[B2] Amiraslanov A. Y. U. Artyukhina E. A. Revishvili A. S. (2022). Long-term results of cardiac contractility modulation in patients with chronic heart failure. Vestn. Aritmologii. 29, 17–23. 10.35336/va-2022-1-03

[B3] Bartolucci C. Passini E. Hyttinen J. Paci M. Severi S. (2020). Simulation of the effects of extracellular calcium changes leads to a novel computational model of human ventricular action potential with a revised calcium handling. Front. Physiol. 11, 314. 10.3389/fphys.2020.00314 32351400 PMC7174690

[B4] Blinova K. Dang Q. Millard D. Smith G. Pierson J. Guo L. (2018). International multisite study of human-induced pluripotent stem cell-derived cardiomyocytes for drug proarrhythmic potential assessment. Cell. Rep. 24, 3582–3592. 10.1016/j.celrep.2018.08.079 30257217 PMC6226030

[B5] Blinova K. Schocken D. Patel D. Daluwatte C. Vicente J. Wu J. C. (2019). Clinical trial in a dish: Personalized stem cell-derived cardiomyocyte assay compared with clinical trial results for two QT-prolonging drugs. Clin. Transl. Sci. 12, 687–697. 10.1111/cts.12674 31328865 PMC6853144

[B6] Blinova K. Stohlman J. Krauthamer V. Knapton A. Bloomquist E. Gray R. A. (2014). Acute effects of nonexcitatory electrical stimulation during systole in isolated cardiac myocytes and perfused heart. Physiol. Rep. 2, e12106. 10.14814/phy2.12106 25096553 PMC4246583

[B7] Blinova K. Stohlman J. Vicente J. Chan D. Johannesen L. Hortigon-Vinagre M. P. (2017). Comprehensive translational assessment of human-induced pluripotent stem cell derived cardiomyocytes for evaluating drug-induced arrhythmias. Toxicol. Sci. 155, 234–247. 10.1093/toxsci/kfw200 27701120 PMC6093617

[B8] Brunckhorst C. B. Shemer I. Mika Y. Ben-Haim S. A. Burkhoff D. (2006). Cardiac contractility modulation by non-excitatory currents: Studies in isolated cardiac muscle. Eur. J. Heart Fail. 8, 7–15. 10.1016/j.ejheart.2005.05.011 16202650

[B9] Burkhoff D. Shemer I. Felzen B. Shimizu J. Mika Y. Dickstein M. (2001). Electric currents applied during the refractory period can modulate cardiac contractility *in vitro* and *in vivo* . Heart fail. Rev. 6, 27–34. 10.1023/a:1009851107189 11248765

[B10] Burnett S. D. Blanchette A. D. Chiu W. A. Rusyn I. (2021). Cardiotoxicity hazard and risk characterization of ToxCast chemicals using human induced pluripotent stem cell-derived cardiomyocytes from multiple donors. Chem. Res. Toxicol. 34, 2110–2124. 10.1021/acs.chemrestox.1c00203 34448577 PMC8762671

[B11] Butter C. Rastogi S. Minden H. H. Meyhöfer J. Burkhoff D. Sabbah H. N. (2008). Cardiac contractility modulation electrical signals improve myocardial gene expression in patients with heart failure. J. Am. Coll. Cardiol. 51, 1784–1789. 10.1016/j.jacc.2008.01.036 18452785

[B12] Campbell C. M. Kahwash R. Abraham W. T. (2020). Optimizer Smart in the treatment of moderate-to-severe chronic heart failure. Future Cardiol. 16, 13–25. 10.2217/fca-2019-0044 31825245

[B13] Eng G. Lee B. W. Protas L. Gagliardi M. Brown K. Kass R. S. (2016). Autonomous beating rate adaptation in human stem cell-derived cardiomyocytes. Nat. Commun. 7, 10312. 10.1038/ncomms10312 26785135 PMC4735644

[B14] FDA.GOV (2019). FDA Summary of Safety and Effectiveness Data (SSED). P180036 [Online]. Available at: https://www.accessdata.fda.gov/cdrh_docs/pdf18/P180036b.pdf (Accessed 07 27, 2021).

[B15] Feaster T. K. Cadar A. G. Wang L. Williams C. H. Chun Y. W. Hempel J. (2015). Matrigel mattress: A method for the generation of single contracting human-induced pluripotent stem cell-derived cardiomyocytes. Circ. Res. 117, 995–1000. 10.1161/CIRCRESAHA.115.307580 26429802 PMC4670592

[B16] Feaster T. K. Casciola M. Narkar A. Blinova K. (2021). Acute effects of cardiac contractility modulation on human induced pluripotent stem cell-derived cardiomyocytes. Physiol. Rep. 9, e15085. 10.14814/phy2.15085 34729935 PMC8564440

[B17] Feric N. T. Pallotta I. Singh R. Bogdanowicz D. R. Gustilo M. Chaudhary K. (2019). Engineered cardiac tissues generated in the Biowire™ II: A platform for human-based drug discovery. Toxicol. Sci. 172, 89–97. 10.1093/toxsci/kfz168 31385592 PMC6813749

[B18] Fermini B. Coyne S. T. Coyne K. P. (2018). Clinical trials in a dish: A perspective on the coming revolution in drug development. SLAS Discov. 23, 765–776. 10.1177/2472555218775028 29862873 PMC6104197

[B19] Gintant G. Kaushik E. P. Feaster T. Stoelzle-Feix S. Kanda Y. Osada T. (2020). Repolarization studies using human stem cell-derived cardiomyocytes: Validation studies and best practice recommendations. Regul. Toxicol. Pharmacol. 117, 104756. 10.1016/j.yrtph.2020.104756 32822771

[B20] Harris K. Aylott M. Cui Y. Louttit J. B. Mcmahon N. C. Sridhar A. (2013). Comparison of electrophysiological data from human-induced pluripotent stem cell-derived cardiomyocytes to functional preclinical safety assays. Toxicol. Sci. 134, 412–426. 10.1093/toxsci/kft113 23690542

[B21] Hesselson A. B. Hesselson H. H. Leung S. Vaidya G. (2022). Normalization of ventricular function after cardiac contractility modulation in noncompaction cardiomyopathy heterozygous positive for a pathologic TTN gene variant. Hear. Case Rep. 8, 449–452. 10.1016/j.hrcr.2022.03.016 PMC9237375 35774200

[B22] Huethorst E. Mortensen P. Simitev R. D. Gao H. Pohjolainen L. Talman V. (2022). Conventional rigid 2D substrates cause complex contractile signals in monolayers of human induced pluripotent stem cell-derived cardiomyocytes. J. Physiol. 600, 483–507. 10.1113/JP282228 34761809 PMC9299844

[B23] Hwang H. S. Kryshtal D. O. Feaster T. K. Sanchez-Freire V. Zhang J. Kamp T. J. (2015). Comparable calcium handling of human iPSC-derived cardiomyocytes generated by multiple laboratories. J. Mol. Cell. Cardiol. 85, 79–88. 10.1016/j.yjmcc.2015.05.003 25982839 PMC4530041

[B24] IMPULSEDYNAMICS (2018). OPTIMIZER® smart implantable pulse generator instructions for use. [Online]. Available at: https://impulse-dynamics.com/wp-content/uploads/2020/05/13-290-008-01-US-Rev-01-OPT-Smart-IPG-IFU.pdf .

[B25] IMPULSEDYNAMICS (2019). OPTIMIZER™ smart mini implantable pulse GeneratorINSTRUCTIONS for use. [Online]. Available at: https://impulse-dynamics.com/global/wp-content/uploads/sites/2/2021/02/13-290-011-EU-Rev-00-OPTIMIZER-Smart-Mini-IPG-IFU-EU.pdf (Accessed 02 2021, 19).

[B26] Korner A. Mosqueira M. Hecker M. Ullrich N. D. (2021). Substrate stiffness influences structural and functional remodeling in induced pluripotent stem cell-derived cardiomyocytes. Front. Physiol. 12, 710619. 10.3389/fphys.2021.710619 34489730 PMC8416903

[B27] Kuschyk J. Kloppe A. Schmidt-Schweda S. Bonnemeier H. Rousso B. Roger S. (2017). Cardiac contractility modulation: A technical guide for device implantation. Rev. Cardiovasc. Med. 18, 1–13. 10.3909/ricm0825 28509888

[B28] Lawo T. Borggrefe M. Butter C. Hindricks G. Schmidinger H. Mika Y. (2005). Electrical signals applied during the absolute refractory period: An investigational treatment for advanced heart failure in patients with normal QRS duration. J. Am. Coll. Cardiol. 46, 2229–2236. 10.1016/j.jacc.2005.05.093 16360051

[B29] Ma J. Guo L. Fiene S. J. Anson B. D. Thomson J. A. Kamp T. J. (2011). High purity human-induced pluripotent stem cell-derived cardiomyocytes: Electrophysiological properties of action potentials and ionic currents. Am. J. Physiol. Heart Circ. Physiol. 301, H2006–H2017. 10.1152/ajpheart.00694.2011 21890694 PMC4116414

[B30] Majid Q. A. Fricker A. T. R. Gregory D. A. Davidenko N. Hernandez Cruz O. Jabbour R. J. (2020). Natural biomaterials for cardiac tissue engineering: A highly biocompatible solution. Front. Cardiovasc. Med. 7, 554597. 10.3389/fcvm.2020.554597 33195451 PMC7644890

[B31] Mastoris I. Spall H. Sheldon S. H. Pimentel R. C. Steinkamp L. Shah Z. (2021). Emerging implantable-device technology for patients at the intersection of electrophysiology and heart failure interdisciplinary care. J. Card. Fail. 28, 991–1015. 10.1016/j.cardfail.2021.11.006 34774748

[B32] Meyer T. Tiburcy M. Zimmermann W. H. (2019). Cardiac macrotissues-on-a-plate models for phenotypic drug screens. Adv. Drug Deliv. Rev. 140, 93–100. 10.1016/j.addr.2019.03.002 30902615

[B33] Mohri S. He K. L. Dickstein M. Mika Y. Shimizu J. Shemer I. (2002). Cardiac contractility modulation by electric currents applied during the refractory period. Am. J. Physiol. Heart Circ. Physiol. 282, H1642–H1647. 10.1152/ajpheart.00959.2001 11959626

[B34] Mohri S. Shimizu J. Mika Y. Shemer I. Wang J. Ben-Haim S. (2003). Electric currents applied during refractory period enhance contractility and systolic calcium in the ferret heart. Am. J. Physiol. Heart Circ. Physiol. 284, H1119–H1123. 10.1152/ajpheart.00378.2002 12446280

[B35] Morita H. Suzuki G. Haddad W. Mika Y. Tanhehco E. J. Sharov V. G. (2003). Cardiac contractility modulation with nonexcitatory electric signals improves left ventricular function in dogs with chronic heart failure. J. Card. Fail. 9, 69–75. 10.1054/jcaf.2003.8 12612875

[B36] Nagele H. Behrens S. Eisermann C. (2008). Cardiac contractility modulation in non-responders to cardiac resynchronization therapy. Europace 10, 1375–1380. 10.1093/europace/eun257 18776196

[B37] Narkar A. Feaster T. K. Casciola M. Blinova K. (2022). Human *in vitro* neurocardiac coculture (ivNCC) assay development for evaluating cardiac contractility modulation. Physiol. Rep. 10 e15498 36325586 10.14814/phy2.15498 PMC9630755

[B38] Narkar A. Willard J. M. Blinova K. (2022). Chronic cardiotoxicity assays using human induced pluripotent stem cell-derived cardiomyocytes (hiPSC-CMs). Int. J. Mol. Sci. 23, 3199. 10.3390/ijms23063199 35328619 PMC8953833

[B39] Pappone C. Rosanio S. Burkhoff D. Mika Y. Vicedomini G. Augello G. (2002). Cardiac contractility modulation by electric currents applied during the refractory period in patients with heart failure secondary to ischemic or idiopathic dilated cardiomyopathy. Am. J. Cardiol. 90, 1307–1313. 10.1016/s0002-9149(02)02868-0 12480039

[B40] Patel D. Stohlman J. Dang Q. Strauss D. G. Blinova K. (2019). Assessment of proarrhythmic potential of drugs in optogenetically paced induced pluripotent stem cell-derived cardiomyocytes. Toxicol. Sci. 170, 167–179. 10.1093/toxsci/kfz076 30912807

[B41] Qu Y. Feric N. Pallotta I. Singh R. Sobbi R. Vargas H. M. (2020). Inotropic assessment in engineered 3D cardiac tissues using human induced pluripotent stem cell-derived cardiomyocytes in the Biowire(TM) II platform. J. Pharmacol. Toxicol. Methods 105, 106886. 10.1016/j.vascn.2020.106886 32629159

[B42] Ribeiro M. C. Tertoolen L. G. Guadix J. A. Bellin M. Kosmidis G. D'Aniello C. (2015). Functional maturation of human pluripotent stem cell derived cardiomyocytes *in vitro*--correlation between contraction force and electrophysiology. Biomaterials 51, 138–150. 10.1016/j.biomaterials.2015.01.067 25771005

[B43] Ronaldson-Bouchard K. Ma S. P. Yeager K. Chen T. Song L. Sirabella D. (2018). Advanced maturation of human cardiac tissue grown from pluripotent stem cells. Nature 556, 239–243. 10.1038/s41586-018-0016-3 29618819 PMC5895513

[B44] Sabbah H. N. Haddad W. Mika Y. Nass O. Aviv R. Sharov V. G. (2001). Cardiac contractility modulation with the impulse dynamics signal: Studies in dogs with chronic heart failure. Heart fail. Rev. 6, 45–53. 10.1023/a:1009855208097 11248767

[B45] Sala L. van Meer B. J. Tertoolen L. G. J. Bakkers J. Bellin M. Davis R. P. (2018). Musclemotion: A versatile open software tool to quantify cardiomyocyte and cardiac muscle contraction *in vitro* and *in vivo* . Circ. Res. 122, e5–e16. 10.1161/CIRCRESAHA.117.312067 29282212 PMC5805275

[B46] Saleem U. Mannhardt I. Braren I. Denning C. Eschenhagen T. Hansen A. (2020a). Force and calcium transients analysis in human engineered heart tissues reveals positive force-frequency relation at physiological frequency. Stem Cell. Rep. 14, 312–324. 10.1016/j.stemcr.2019.12.011 PMC7013237 31956082

[B47] Saleem U. van Meer B. J. Katili P. A. Yusof N. A. N. M. Mannhardt I. Garcia A. K. (2020b). Blinded, multicenter evaluation of drug-induced changes in contractility using human-induced pluripotent stem cell-derived cardiomyocytes. Toxicol. Sci. 176, 103–123. 10.1093/toxsci/kfaa058 32421822 PMC7357169

[B48] Schechtman L. M. (2002). Implementation of the 3Rs (refinement, reduction, and replacement): Validation and regulatory acceptance considerations for alternative toxicological test methods. ILAR J. 43, S85–S94. 10.1093/ilar.43.suppl_1.s85 12388858

[B49] Stix G. Borggrefe M. Wolpert C. Hindricks G. Kottkamp H. Bocker D. (2004). Chronic electrical stimulation during the absolute refractory period of the myocardium improves severe heart failure. Eur. Heart J. 25, 650–655. 10.1016/j.ehj.2004.02.027 15084369

[B50] Strauss D. G. Blinova K. (2017). Clinical trials in a dish. Trends Pharmacol. Sci. 38, 4–7. 10.1016/j.tips.2016.10.009 27876286 PMC5379998

[B51] SUMMARY OF SAFETY AND EFFECTIVENESS DATA (2021). (SSED) [Online]. Available at: https://www.accessdata.fda.gov/cdrh_docs/pdf18/P180036B.pdf .

[B52] Sun N. Yazawa M. Liu J. Han L. Sanchez-Freire V. Abilez O. J. (2012). Patient-specific induced pluripotent stem cells as a model for familial dilated cardiomyopathy. Sci. Transl. Med. 4, 130ra47. 10.1126/scitranslmed.3003552 PMC3657516 22517884

[B53] Tint D. Florea R. Micu S. (2019). New generation cardiac contractility modulation device-filling the gap in heart failure treatment. J. Clin. Med. 8, E588. 10.3390/jcm8050588 PMC6572164 31035648

[B54] Tsan Y. C. Depalma S. J. Zhao Y. T. Capilnasiu A. Wu Y. W. Elder B. (2021). Physiologic biomechanics enhance reproducible contractile development in a stem cell derived cardiac muscle platform. Nat. Commun. 12, 6167. 10.1038/s41467-021-26496-1 34697315 PMC8546060

[B55] Veldhuizen J. Migrino R. Q. Nikkhah M. (2019). Three-dimensional microengineered models of human cardiac diseases. J. Biol. Eng. 13, 29. 10.1186/s13036-019-0155-6 30988697 PMC6448321

[B56] Wang L. Kim K. Parikh S. Cadar A. G. Bersell K. R. He H. (2018). Hypertrophic cardiomyopathy-linked mutation in troponin T causes myofibrillar disarray and pro-arrhythmic action potential changes in human iPSC cardiomyocytes. J. Mol. Cell. Cardiol. 114, 320–327. 10.1016/j.yjmcc.2017.12.002 29217433 PMC5800960

[B57] Winter J. Brack K. E. Coote J. H. Ng G. A. (2014). Cardiac contractility modulation increases action potential duration dispersion and decreases ventricular fibrillation threshold via β1-adrenoceptor activation in the crystalloid perfused normal rabbit heart. Int. J. Cardiol. 172, 144–154. 10.1016/j.ijcard.2013.12.184 24456882 PMC3978661

[B58] Winter J. Brack K. E. Ng G. A. (2011). The acute inotropic effects of cardiac contractility modulation (CCM) are associated with action potential duration shortening and mediated by β1-adrenoceptor signalling. J. Mol. Cell. Cardiol. 51, 252–262. 10.1016/j.yjmcc.2011.04.010 21557948 PMC3176912

[B59] Yang X. Ribeiro A. J. S. Pang L. Strauss D. G. (2022). Use of human iPSC-CMs in nonclinical regulatory studies for cardiac safety assessment. Toxicol. Sci., kfac095. 10.1093/toxsci/kfac095 36099065

[B60] Yee J. (2008). “xPharm: The comprehensive pharmacology reference,” in xPharm: The comprehensive pharmacology reference. Editor BYLUND S. J. E. A. D. B. (Elsevier).

