# Supplementary material for: Acute effects of cardiac contractility modulation stimulation in conventional 2D and 3D human induced pluripotent stem cell-derived cardiomyocyte models
Source: Front Physiol. 2022 Nov 10;13:1023563. doi: 10.3389/fphys.2022.1023563 (PMC9686332; doi:10.3389/fphys.2022.1023563)
Supplement: Supplementary file 1 [file Table1.DOCX]

Supplementary Material

# Supplemental Tables:

| Supplemental Table 1. Range of CCM Pulse Parameters | | |
| --- | --- | --- |
| CCM Pulse Parameter | Clinical CCM Range^1,2^ | Values Tested |
| Pulse Number | 1 - 3 | 1, 2, 3 |
| Pulse Delay (ms) | 3 - 140 | 3, 15, 30, 60, 90, 120, 140, 160 |
| Pulse Duration (ms) | 5.14 - 6.6 | 4.5, 5, 5.14, 6, 6.6, 7 |
| Pulse Amplitude (V) | 4.0 - 7.5 | 1, 2.5, 5, 7.5, 10 |

^1.2 (ImpulseDynamics, 2018, ImpulseDynamics, 2019)^

| Supplemental Table 2. Baseline Contractile Properties | | |
| --- | --- | --- |
| Parameter | 2D Monolayer hiPSC-CM | 3D ECT |
| Peak Amplitude (a.u.) /Force (µN) | 1378 ± 102.7 | 48.4 ± 2.2 |
| Contraction Duration 50% (ms) | 457.4 ± 11.6 | 68.6 ± 1.4 |
| Contraction Slope (a.u./s) /Force (µN/s) | 18080.3 ± 1724.1 | 1095 ± 56.2 |
| Relaxation Slope (a.u./s) /Force (µN/s) | -7745.5 ± 684.6 | -551.7 ± 24.6 |
| Time to Peak (ms) | 251.3 ± 20.4 | 68.4 ± 1.9 |
| Time to Baseline 90% (ms) | 391.7 ± 18.5 | 120.3 ± 2.0 |
| BPM | 60 | 60 |
| N | 16 | 18^#^ |
| ^#^3 total ECTs, 18 repeated measurements |  |  |

| Supplemental Table 3. CCM Direct Contraction Stress in 3D ECTs | | | | | | | |
| --- | --- | --- | --- | --- | --- | --- | --- |
| Delay (ms) | Stress (mN/mm^2^) | Duration (ms) | Stress (mN/mm^2^) | Amplitude (V) | Stress (mN/mm^2^) | Pulse Number | Stress (mN/mm^2^) |
| 3 | 0.57 ± 0.04 | 4.5 | 0.91 ± 0.11 | 1 | 0.74 ± 0.08 | 1 | 0.68 ± 0.10 |
| 15 | 0.81 ± 0.08 | 5.14 | 0.99 ± 0.13 | 2.5 | 0.73 ± 0.13 | 2 | 1.01 ± 0.10 |
| 30 | 1.03 ± 0.12 | 5.5 | 1.10 ± 0.07 | 5 | 0.74 ± 0.13 | 3 | 1.16 ± 0.08 |
| 60 | 1.25 ± 0.11 | 6 | 1.13 ± 0.07 | 7.5 | 1.08 ± 0.11 |  |  |
| 90 | 1.41 ± 0.15 | 6.6 | 1.22 ± 0.09 | 10 | 1.69 ± 0.08 |  |  |
| 120 | 1.58 ± 0.18 | 7 | 1.22 ± 0.14 |  |  |  |  |
| 140 | 1.61 ± 0.16 |  |  |  |  |  |  |
| 160 | 1.75 ± 0.17 |  |  |  |  |  |  |

#
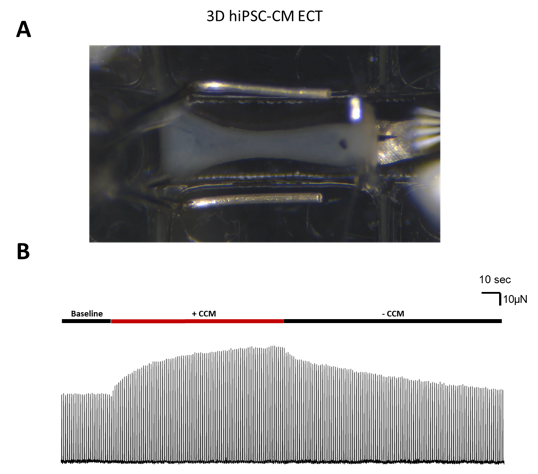
Supplemental Figure 1:

Immobilized end

Force transducer

**A**


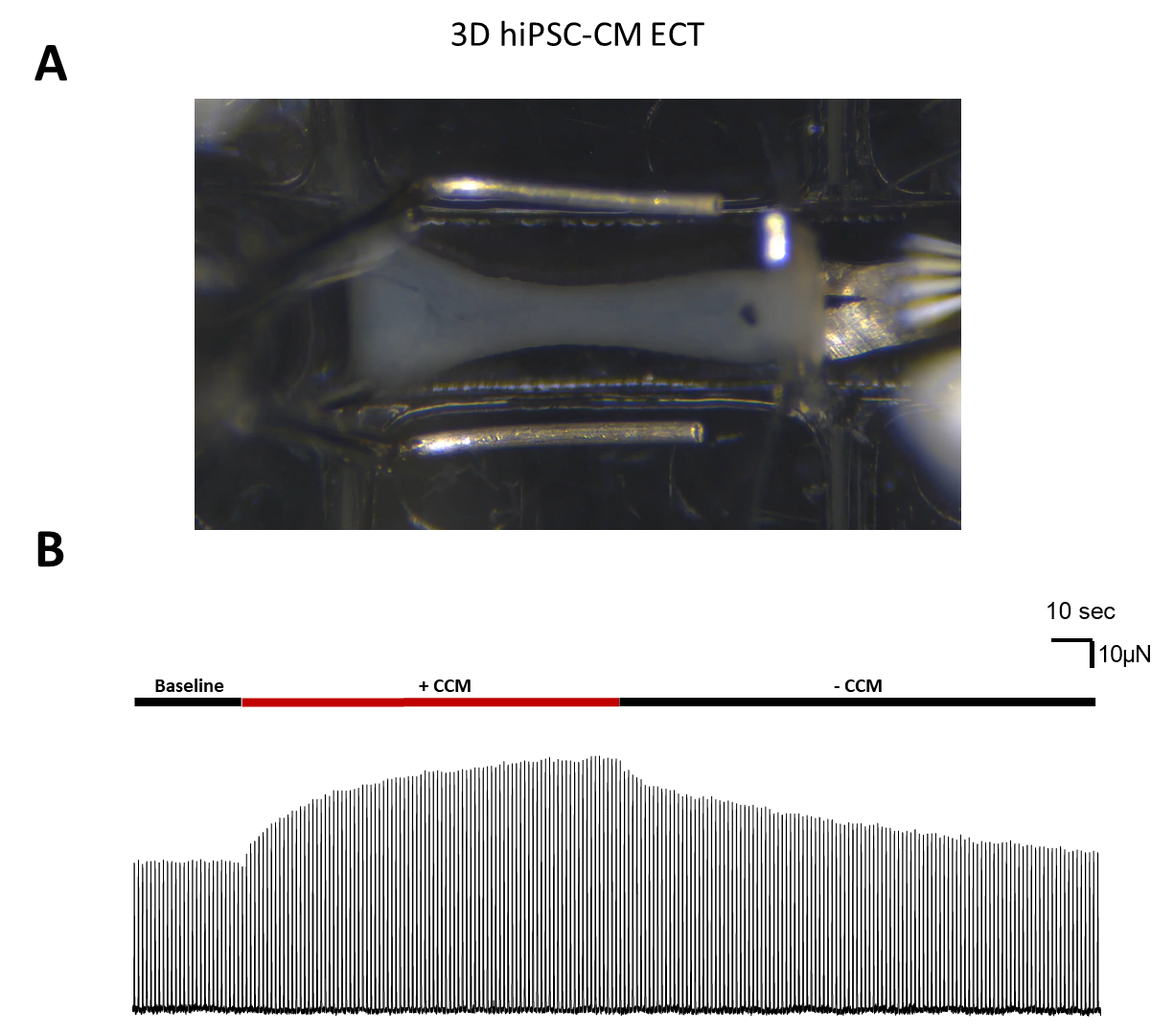


Stimulating electrodes

**B**

**C**

**Supplemental Figure 1.** Effect of CCM on 3D ECT Contraction. **(A)** Cartoon showing the setup used to measure force in the 3D tissues. The polymer was cut at one end of the tissue to free the tissue and the other end was immobilized using platinum wire. The free end of the tissue was attached to a force transducer. **(B)** Video of baseline (i.e., field stimulation pacing, 1 Hz), CCM (7.5 V) at 28 seconds, and recovery. **(C)** Corresponding contraction recording.

##### Supplemental Figure 2:


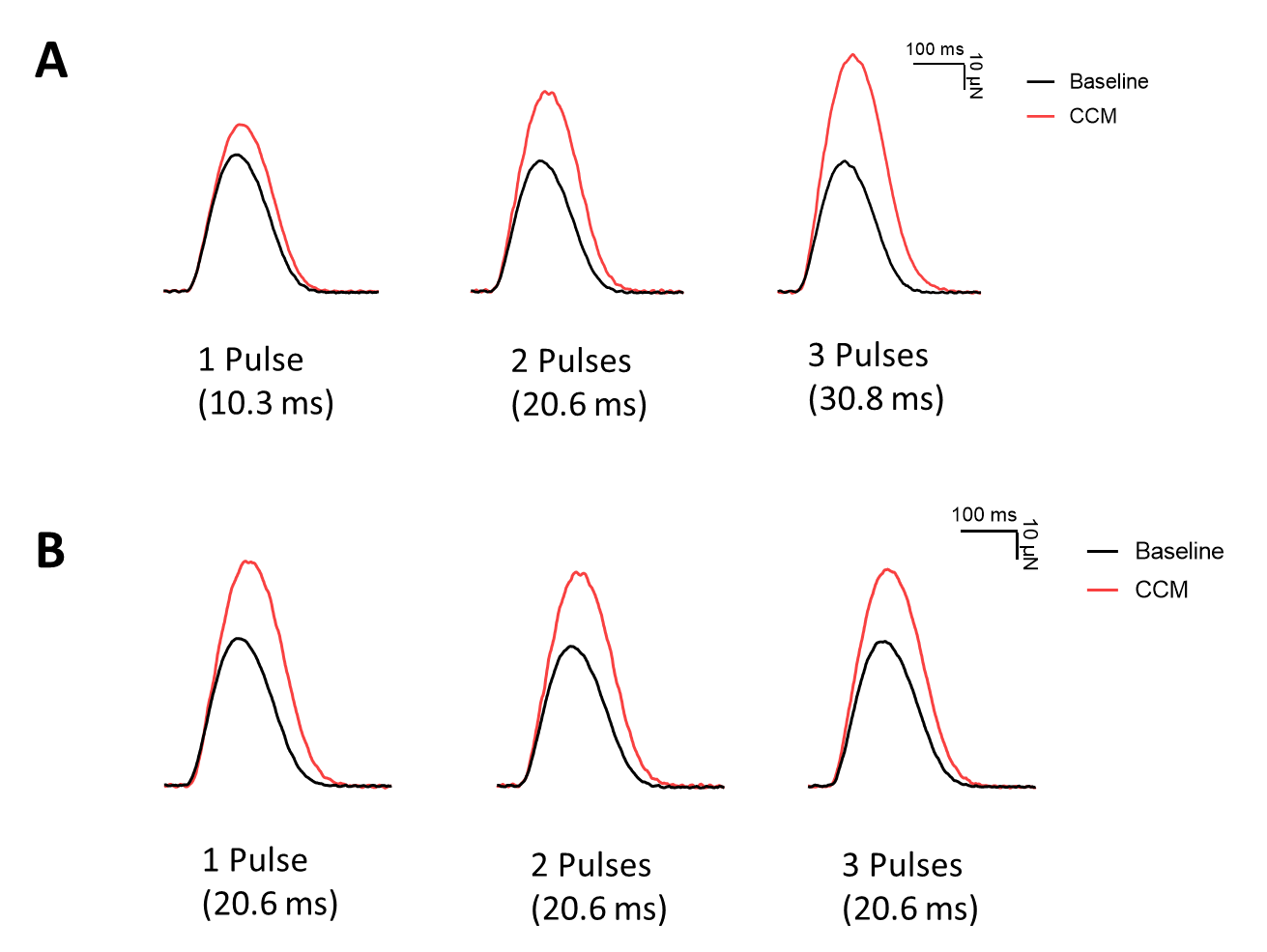


**Supplemental Figure 2.** Effect of CCM pulse number with variable and fixed total duration time in 3D ECTs. **(A)** Representative contraction traces for CCM with 1, 2, and 3 pulses with variable total duration time of 10.3 ms, 20.6 ms and 30.8 ms, respectively. **(B)** Representative contraction traces for CCM with 1, 2, and 3 pulse with fixed total duration time of 20.6 ms, n = 1.

#####
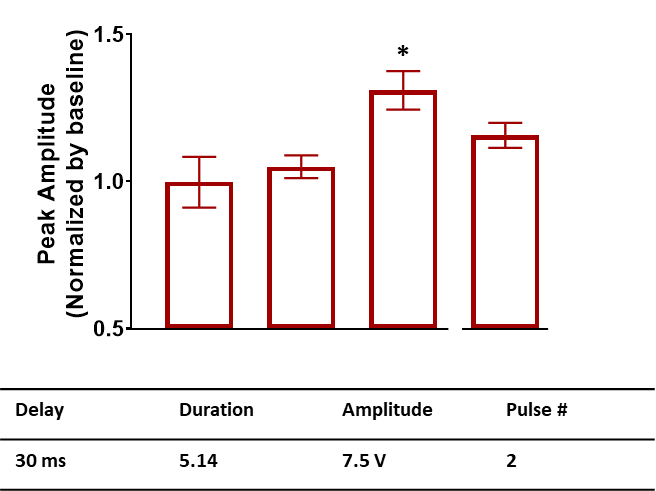
Supplemental Figure 3:

**Supplemental Figure 3.** Effects of CCM on various hiPSC-CM models with submaximal extracellular calcium. Effect of standard clinical CCM signal (i.e., 2 biphasic pulse, 7.5 V, 5.14 ms duration, 30 ms delay) on conventional 2D (stiff substrate), 2D (flexible substrate), and 3D ECT hiPSC-CM models. Extracellular Ca concentration [0.5 mM]. Summary data graphs. Data are mean ± SEM. n = 3 - 23 per group. ^*^P< 0.05 using One-Way ANOVA Kruskal–Wallis comparison. 2D Stiff and 3D ECT data are transformed from figure 3. 2D flex (10 V) data are transformed from (Feaster et al., 2021),

**
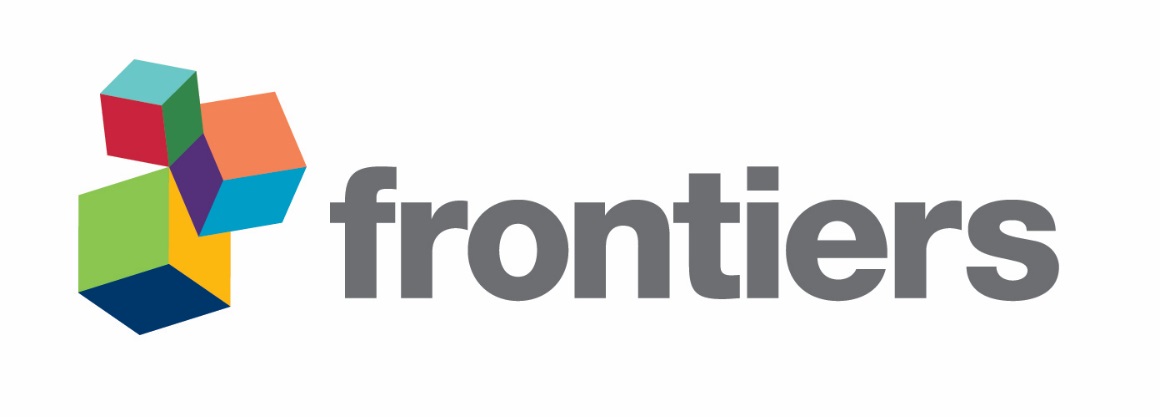
**

# References

FEASTER, T. K., CASCIOLA, M., NARKAR, A. & BLINOVA, K. 2021. Acute effects of cardiac contractility modulation on human induced pluripotent stem cell-derived cardiomyocytes. *Physiol Rep,* 9**,** e15085.

IMPULSEDYNAMICS. 2018. *OPTIMIZER® Smart Implantable Pulse Generator INSTRUCTIONS FOR USE* [Online]. Available: <https://impulse-dynamics.com/wp-content/uploads/2020/05/13-290-008-01-US-Rev-01-OPT-Smart-IPG-IFU.pdf> [Accessed].

IMPULSEDYNAMICS. 2019. *OPTIMIZER™ Smart Mini Implantable Pulse GeneratorINSTRUCTIONS FOR USE* [Online]. Available: <https://impulse-dynamics.com/global/wp-content/uploads/sites/2/2021/02/13-290-011-EU-Rev-00-OPTIMIZER-Smart-Mini-IPG-IFU-EU.pdf> [Accessed 2021-02-19].
